# Social participation in the unified health system of Brazil: an exploratory study on the adequacy of health councils to resolution 453/2012

**DOI:** 10.1186/s12913-021-07161-1

**Published:** 2021-10-19

**Authors:** Rita de Cássia Costa da Silva, Maykon Anderson Pires de Novais, Paola Zucchi

**Affiliations:** 1 Programa de Pós-Graduação em Gestão e Informática em Saúde, Escola Paulista de Medicina, Universidade Federal de São Paulo, São Paulo, SP Brazil; 2 Programa de Pós-Graduação em Gestão e Informática em Saúde, Disciplina de Economia e Gestão em Saúde, Escola Paulista de Medicina, Universidade Federal de São Paulo, São Paulo, SP Brazil; 3 Programa de Pós-Graduação em Gestão e Informática em Saúde, Disciplina de Economia e Gestão em Saúde, Escola Paulista de Medicina, Universidade Federal de São Paulo, São Paulo, SP Brazil

**Keywords:** Community participation, Social participation, Health councils, Unified health system, Legal standards, Brazil

## Abstract

**Introduction:**

Social participation is one of the guidelines of the Brazilian health system. Health councils are collegiate instances of participation established by Law 8.142/90. The most recent legal regulation for council organization and functioning was established through Resolution 453/2021. The institution of health councils has a permanent and deliberative nature to act in the formulation, deliberation and control of health policy implementation, including in economic and financial aspects.

**Objective:**

To evaluate the compliance of health councils with the directives for the establishment, restructuring and operation of the councils from Brazil, based on Resolution 453/2012.

**Methods:**

An exploratory, descriptive study that used the Health Council Monitoring System as a data source. Qualitative variables were selected to identify the characteristics related to the councils’ establishment (legal instruments for establishment), the strategies adopted for restructuring (budget allocation, existence of an executive secretariat, provision of a dedicated office) and the characteristics of the health councils’ operation (frequency of regular meetings, existence of a board of directors, the election of the board of directors).

**Results:**

The study analyzed three groups of characteristics related to the constitution, strategies adopted for restructuring and the functioning of the councils. Regarding the constitution of the councils, the findings revealed that the vast majority was constituted in accordance with the legislation and, therefore, is in compliance with Resolution 453/2021. In the second group of characteristics that describe the restructuring of councils, the study found that less than half of registered councils are in compliance with the standard. And, finally, in the third group of characteristics, it was found that the boards have adopted different frequencies for regular meetings and approximately 50% of the boards studied have a board of directors.

**Conclusions:**

The councils still do not meet the minimum conditions necessary to fulfil their role in the Unified Health System (SUS), as stipulated in Resolution 453/2021. This situation requires monitoring by public oversight agencies. Despite the increase in popular participation with the creation of the health councils, this study demonstrated that most councils still do not meet the minimum conditions for monitoring public health policy. The improvement of the Health Councils Monitoring System (SIACS) to become an instrument for monitoring the councils, with the definition of goals and results, may contribute to the organization of the councils and, therefore, to the realization of social participation in Brazil.

## Introduction

Brazil’s public health system was conceived in the 1980s in the midst of popular movements calling for the democratization of the state and policies guaranteeing basic human rights [1]. Precarious health conditions and limitations of the population’s access to the network of health care services drove social pressure for change. At the time, the country was experiencing a re-democratization that resulted in the drafting of a new Constitution, an expression of popular demands [2]. The enactment of the 1988 Federal Constitution marked the end of the long years of military rule and encouraged the creation of public policy councils, with increased popular participation.

The Brazilian Health Reform Movement (MRSB, for its acronym in Portuguese) was the key driver of transformation that led to the attainment of the right to health, which was established in the constitutional text as a universal right and an obligation of the state [3]. The Eighth National Health Conference, held in 1986, was a historic milestone in the creation of the Unified Heath System (SUS). The Conference included broad participation from civil society, which at the time was unprecedented.

In addition to discussing health conditions in Brazil, the Conference identified the need to create mechanisms to include the population in the development and monitoring of health policy in municipalities, states, and the whole country. The SUS was enshrined in the Constitution and later regulated by the 1990 laws 8080 and 8142. The organizational principles of the new health system were based on community participation, decentralization, and a hierarchy of health care services [4].

The idea of social participation within the SUS is aligned with the consensus among advocates for participatory democracy that has been observed since the 1990s: public management may perform better when the population is involved [5]. The institutionalization of social participation in the SUS resulted in the mandatory creation of two collective bodies in each sphere of government: conferences and health councils.

Pursuant to law 8142/90, which stipulates community participation in the management of the SUS, health councils became mandatory, and they began to be established in Brazil in the early 1990s [4]. The councils are legitimate bodies for citizen participation. According to data from the Health Council Monitoring System (SIACS, for its acronym in Portuguese), 5631 health councils have been established across all counties, states, and Brasília, the Federal District. A crucial factor for the rapid spread of those councils was financial transfers from the federal government to states and counties that were linked to the creation of health councils and other aspects, such as the establishment of a health fund, a health insurance scheme and a committee to develop career plans, positions and salaries for health professionals in the SUS [6].

Health councils are permanent and deliberative bodies that formulate, deliberate and control the execution of health policy, including economic and financial aspects. The composition of this collegiate body should be parity and should guarantee the presence of representatives from of the population that uses the public health system, as well as workers, managers, and service providers [6]. This equality is intended to guarantee that users participate as equals with other representatives to deliberate the health policy. The councils and the health conferences should thus comprise 50% users, 25% workers and 25% SUS managers and service providers. Participation is voluntary, and therefore, no compensation can be provided for the council members’ involvement in the council’s activities [5].

The institutionalization of participation through the councils, however, was not sufficient to guarantee effective social control over public policies. On the contrary, studies indicate that it produced complacency, making participation “ritualized and lacking in spontaneity” [5]. The challenges to participation are ongoing, particularly now in Brazil, when there are major setbacks for democratic participation in government.

Among the challenges the health councils face is the need for institutional support that guarantees a structure for their operation. The presence of administrative and financial support demonstrates that councils have the resources needed to carry out their duties properly. Yet, if those conditions are not present, health councils may represent a quantitative gain (because they are present in all counties, states and the Federal District) but will not meet the requisite qualifications for deliberating and monitoring relevant health policies [7].

Regarding the infrastructure necessary for health councils, the final report of the 10th National Health Conference, held in 1996, highlighted criteria that include budget allocations for the council, a permanent physical space, and technical support for carrying out activities. It also emphasized the need to provide educational activities on an ongoing basis [8].

The National Health Council showed concern regarding the structure and operation of the councils when it approved Resolution 453/2012. Although some analyses have pointed out weaknesses and gaps in that resolution, it considered broad debates and demands from health councils to improve the social control process in the SUS [9].

Resolution 453/2012 is thus the most recent infra-constitutional instrument that mandates the restructuring of county, state and Federal District health councils in Brazil. Some of the most prominent aspects of the establishment and operation of the councils are that they should be established by law and have complete administrative autonomy. They should also have their own budget allocation, financial autonomy, and an executive secretariat with the necessary infrastructure and technical support [10].

This study recognizes that the establishment of spaces for public participation in the management of the public health system, as in the case of the health councils, is an achievement of social movements in Brazil. Nonetheless, despite the existence of those spaces, the population’s direct participation in the development of public health policies is still relatively limited [11]. Despite the institutionalization of social participation in the SUS, many councils still do not have adequate conditions and infrastructure to operate properly. Hence, studies that show the health councils’ compliance with the legal regulations that govern their establishment, restructuring and operation, which are necessary conditions for the qualified performance of health council members, are important. Previous studies indicated that a lack of support and infrastructure prevents councils from carrying out their duties [11, 12]. This study thus sought to reveal aspects related to the establishment, restructuring and operation of the councils, as proposed in Resolution 453/2012, with the understanding that a minimum structure is necessary for participation and social control to be fully achieved in the SUS.

Considering that the councils need a physical and administrative structure to operate properly, lest they become spaces for legitimizing the powers that be, this study aimed to evaluate the health councils’ compliance with the directives for their establishment, restructuring and operation based on Resolution 453/2012 of the National Health Council.

## Methods

This is an exploratory, descriptive, comparative study of data reported to the SIACS. The population comprised all health councils in Brazil. The study used a secondary data source collected from May to August 2017 from the website http://conselho.saude.gov.br/web_siacs/index.html, which hosts the SIACS. The SIACS is a public online database of the National Health Council. The system contains data related to the establishment, organization, structure, and operation of health councils, among other information. For a health council to meet the eligibility criterion, it had to be included in the SIACS during the data collection period. Health councils that were not registered in the SIACS were excluded from the study.

Based on the recommendations of the National Health Council’s Resolution 453/2012 and the information available in the SIACS, qualitative variables were selected to identify the characteristics of the councils’ establishment (legal instruments for establishment), the strategies adopted for restructuring (budget allocation, existence of an executive secretariat, the presence of a dedicated office) and their operating characteristics (frequency of regular meetings, existence of a board of directors, reports of elections for the board of directors).

The data were recorded for each unit individually and organized in a Microsoft Office Excel spreadsheet. Data were analyzed by geographic region, and descriptive statistics and exploratory analysis were used to determine the relative frequency of the variables.

Resolution 453/2012 was adopted as a reference for the data analysis, making it possible to reveal the number of councils that were and were not compliant with the requirements for their establishment, restructuring and operation.

This study is part of the research project “Analysis of the Current Situation of Health Councils in Brazil,” which was approved by the Research Ethics Committee in resolution 1,424,026.

## Results

The study sample comprised 4742 (84.2%) health councils out of the 5631 that exist in Brazil. The study analyzed three groups of characteristics related to the constitution, strategies adopted for restructuring and the functioning of the councils.

In the first group that deals with the constitution of the councils, analysis revealed that 4543 (95.8%) councils were established by law and are thus compliant with Resolution 453/2012. The 199 (4.2%) councils that are not compliant with the Resolution were created by decrees or ordinances. Among the 4543 councils established by law, the compliance percentage is above 90% (Table 1) in all regions. Acre, in the West Amazon Region, is the only state in the country where all 23 councils were created by law. In contrast, the Federal District, in the Midwest Region, does not have any councils established by law. Of the five existing councils in the Federal District, two were established by decrees and three by ordinances.

**Table 1:** Instrument for establishing councils, 2017

Region	Compliant (law)		Not compliant (decree/ordinance)		Total by region
	N	%	N	%	
Midwest	382	94.5	22	5.5	404
North	344	95.0	18	5.0	362
Northeast	1542	96.3	59	3.7	1601
South	1106	95.7	49	4.3	1155
Southeast	1169	95.8	51	4.2	1220
Overall Total	4543	95.8	199	4.2	4742

Source: SIACS

Regarding to the second group of characteristics that are related to the restructuring of the councils, checked that only 1674 (36.5%) of the health councils had a budget allocation; 2913 (63.5%) did not. The region with the highest percentage of councils with a budget allocation was the North region: 190 (54.1%). In the other regions of Brazil, the percentage is below 42%, and the lowest percentages are in the South (337; 30.1%) and Southeast (374; 31.7%) Regions. 2337 (49.3%) councils have an executive secretariat to advise and support the work of health council members. The number of noncompliant councils was 2405 (50.7%). The North and Northeast regions were noteworthy for having, respectively, more than 64 and 51% of councils without an executive secretariat. In the other regions, the percentage of councils without an executive secretariat ranged between 43 and 50%. Only 1382 (30.1%) councils had their own office spaces. The other 3205 (69.9%) did not have an office. Across all Brazilian regions, the percentage of councils that did not have an is high, ranging between 63 and 79%. Regarding restructuring, there was no information about budget allocation and dedicated headquarters for 155 (3.3%) councils (Table 2).

**Table 2:** Relative distribution of budget allocation, executive secretariat and dedicated headquarters in the health councils, 2017

Region	Budget allocation				Executive secretariat				Dedicated headquarters			
	Yes		No		Yes		No		Yes		No	
	N	%	N	%	N	%	N	%	N	%	N	%
Midwest	138	35.2	254	64.8	197	50.2	207	49.7	144	36.7	248	63.3
North	190	54.1	161	45.8	125	35.6	237	64.4	129	36.7	222	63.2
Northeast	635	41.1	910	58.9	754	48.8	847	51.2	590	38.2	955	61.8
South	337	30.1	783	69.9	636	56.9	519	43.2	227	20.3	893	79.7
Southeast	374	31.7	805	68.3	625	53.0	595	47.0	292	24.8	887	75.2
Total	1674	36.5	2913	63.5	2337	49.3	2405	50.7	1382	30.1	3205	69.9

Source: SIACS

### As for the third group of characteristics that evaluated the functioning aspects of the councils, different frequencies were identified for holding ordinary meetings

**Fig. 1:**
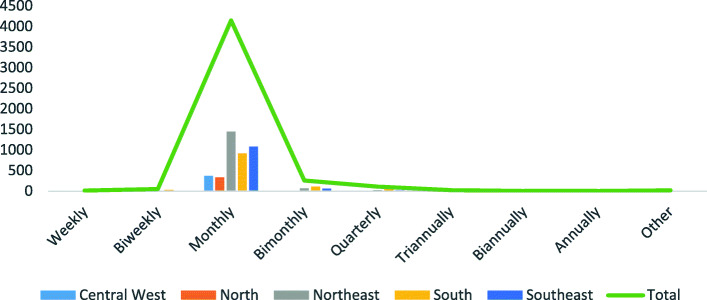
Frequency of regular meetings, Health Councils, 2017. Source: SIACS

The majority, 4146 (87.4%) across all Brazilian regions, were compliant with Resolution 453/2012 and held regular meetings monthly. The other 596 (12.6%) were not compliant and met at the following frequencies: weekly 12 (0.2%), biweekly 52 (1.1%), bimonthly 259 (5.6%), quarterly 108 (2.3%), triannually 22 (0.5%), biannually 3 (0.7%), annually 2 (0.1%) and other 20 (0.4%) (Fig. 1). The councils located in Alagoas, in the Northeast Region, and Amapá, Rondônia and Roraima, in the North Region, were particularly noteworthy, as they were 100% compliant with Resolution 453/2012 with regard to holding regular monthly meetings.

The existence of a board of directors was identified in 2444 (52.8%) councils. Those without a board of directors corresponded to 2188 (47.2%). The North region had the highest percentage of councils with a board of directors (73.2%). In the other regions, the percentage of councils that were compliant with Resolution 453/2012 ranged between 47 and 57%. Regarding the variable “holds elections for the board of directors,” 2326 (50.2%) of the councils were compliant with Resolution 453/2012, while 118 (49.8%) were not (Table 3). Among the 4742 councils in the sample, 118 (2.5%) had no information regarding the frequency of meetings, and 110 (2.3%) had no information regarding the existence of a board of directors and whether elections were held for the board of directors.

**Table 3:** Relative distribution of board of directors and elections for board of directors in the health councils, 2017

Region	Board of directors				Elections for the board of directors			
	Yes		No		Yes		No	
	N	%	N	%	N	%	N	%
Midwest	208	52.3	190	47.7	199	50.0	9	50.0
North	257	73.2	94	26.8	250	71.2	7	28.8
Northeast	711	46.6	848	53.4	678	43.5	34	56.5
South	649	57.1	487	42.9	621	54.7	2	45.3
Southeast	619	52.1	569	47.9	578	48.6	40	51.4
Total	2444	52.8	2188	47.2	2326	50.2	118	49.8

Source: SIACS

The results obtained in this study demonstrate that the effort undertaken by the National Health Council, with the publication of Resolution 453/2012, to guide health councils towards improving their activities did not achieved complete success, especially in aspects related to restructuring. The findings indicate that the edition of the standard, by itself, was not sufficient to fulfill the objectives of consolidating, strengthening, expanding and accelerating the SUS Social Control process, as there was no evidence of homogeneity in the application of Resolution 453/2012 by the health councils. However, this analysis must take into account the limitations of the study, such as the existence of health councils without registration in the SIACS, during the collection period, and the conceptual mismatch with the terminology adopted by Resolution 453/2012, which can lead to different interpretations by health counselors. Furthermore, the use of secondary data is also considered a limiting factor, as it allows little control over the information obtained.

## Discussion

By applying the guiding principles of Resolution 453/2012, we discovered that councils were working towards fulfilling their institutional role in the formulation and oversight of public health policies. Nonetheless, this study shows that health councils across all regions of Brazil were not in compliance with the directives of Resolution 453/2012 regarding their establishment, restructuring and operation.

In countries with a democratic tradition, such as Brazil, laws are created by the National Congress (Senate and House) at the federal level, by State Houses in the states and by City Councils or County Board of Supervisors in Cities and Counties. The establishment of councils created by law is particularly important when considering the principle of legality, according to which no one is obliged to do or refrain from doing anything except pursuant to the law [13]. From this perspective, the operation and establishment of the councils is more strongly guaranteed when councils follow the law.

However, councils established by legal instruments that have a lower impact in the legal hierarchy are more limited in their deliberative character, particularly in situations that are unfavourable to social participation. Decrees, for example, are administrative acts that fall within the exclusive jurisdiction of the heads of the executive branch. Both ordinances and decrees are infra-legal statutes that have less power than laws [14]. Thus, there are a number of different infra-legal regulatory instruments which pose no obstacle to the establishment of councils by a decree or ordinance, according to the provision in Law 8142/90. Resolution 453/2012, however, recognizes only the law as an instrument for establishing councils. This decision conveys the National Health Council concern regarding the sustainability of the councils and the strengthening of participatory democracy within the SUS. It demonstrates the recognition that approval by different legal statutes confers greater or lesser fragility to participatory bodies in the SUS. Recent experiences in Brazil illustrate that spaces for the participation of civil society that are not created by law are more fragile. Presidential Decree 9759, of June 28, 2019, eliminated collegiate bodies in the federal public administration— which were not established by law—without any discussion with civil society. The National Health Council was not affected only because it was created by Law 378 of January 13, 1937.

SUS managers in each government body must provide a budget allocation, an executive secretariat, and offices for the operation of the councils. Those resources promote administrative autonomy for monitoring and supervising health policies. Resolution 453/2012, by approving directives for restructuring the councils, recognizes the need for compliance. Particularly regarding budget allocation, our findings indicate a serious situation in most of the health councils. When health councils do not have financial autonomy for carrying out their activities, they remain subordinate to health departments. In such cases, the supervisory role of the councils is compromised due to the dependence between supervisory bodies (health councils) and those responsible for executing health policy (health departments).

The low percentage of councils that had an executive secretariat and their own offices is also concerning. Our results show administrative dependence of the councils on the executive branch for carrying out their duties, which compromises effective social control over health policies. This limitation must be overcome or the councils will not be able to achieve the “[...] democratic ideal of expanding the public sphere, enabled by participation” [15]. Nonetheless, the existence of an executive secretariat has improved over the years compared to findings of a previous study [16]. There is evidence of a slow participatory process, which suggests both a lack of knowledge by the councils about the directives proposed in Resolution 453/2012 and a low level of investment by SUS managers in strengthening participatory bodies.

The analysis of data regarding the restructuring of the councils indicates that there were not sufficient structural conditions to support decision-making and the monitoring of health activities and services. The lack of budget allocation, an executive secretariat and dedicated headquarters are constraints that limit health council members’ performance in monitoring and controlling health policies. This situation demands intervention and ongoing monitoring by public control agencies to strengthen social participation in the SUS. A study published in 2014 also indicated that a lack of basic resources and equipment compromises effective social control in the SUS [15].

Regular council meetings should take place at least once a month and, in exceptional circumstances, whenever necessary [10]. Regular meetings and an administrative structure are considered evidence of active engagement by health councils, guaranteeing the continuity of debates related to the development of the health policies [17]. The large number of councils that held monthly regular meetings suggests diligent monitoring, with the potential to influence decision-making in states, counties and municipalities, and the Federal District. In that regard, the observed situation is similar to previous findings, demonstrating that regular meeting frequency is the National Health Council recommendation with which the councils were most compliant [16]. However, it is also important to consider the subjects that are addressed in those meetings, which was not the object of this study.

The operating dynamic of the councils also requires a board of directors with equal representation to guarantee a balance of power in the performance of activities. Similarly, the holding of elections for the board of directors reinforces the democratic principles that govern participatory bodies in the Brazilian health system. Our study also revealed that some councils were not coordinated by a board of directors. It is necessary to democratize the internal processes of the councils to guarantee equality in decision-making and expand the dialogue and representativeness of the segments that comprise them.

The sample in our study was representative, and the data were collected from a national database that is publicly accessible. However, one limitation of the study is that although the SIACS has been available since 2012, during the collection period there were health councils for which no information existed available in the system. The use of secondary data is also a limitation as it does not allow for control over the data. Although the SIACS and Resolution 453/2012 were implemented in the same year, there is no consistency in the terminology used, which can lead to different interpretations by the councils when they submit information requested by the SIACS.

## Conclusions

In publishing Resolution 453/2012, the National Health Council reaffirmed that the effectiveness of social control in the SUS is directly related to the organizational capability of the health councils, which must have the support of local managers to guarantee their autonomy. This study found that despite the expansion of popular participation through the creation of health councils, most of the councils still do not meet the minimum conditions necessary for monitoring public health policies.

Our study mapped out the landscape of health councils based on the requirements indicated in Resolution 453/2012 and demonstrated that a significant number of councils are not compliant, indicating the need for coordinated monitoring by National, State, and County Councils. It shows that although health councils have existed for more than three decades, there were still councils that were not compliant with the National Health Council recommendations. Among the monitoring actions that can be implemented, it is suggested to define deadlines for the adequacy of councils that have not yet complied with the provisions of Resolution 453/2012, as well as quarterly monitoring of the Siacs by the National Health Council, with publication of reports consolidated on the situation of health councils in Brazil. In fact, no evidence was identified that the SIACS has been regularly used to monitor the councils, which characterizes it more as an information repository. In the last two decades, the Brazilian health system has achieved important advances in health informatization processes, with the implementation of computerized systems for evaluation and monitoring in the various areas of planning and health care. This advance, in our view, can also reach the health councils, contributing a lot to the realization of social participation in Brazil and in the world, through the improvement of Siacs. Thus, we would, in fact, have a monitoring system, with the definition of goals and results to be achieved by the councils, in addition to the dissemination of regular consolidated reports, which is not yet a reality in Brazill.

## Data Availability

The datasets used and/or analysed during the current study are available from the corresponding author upon reasonable request.
